# Children's Numerical Estimation Is Biased by Male Informants More Than Female Informants

**DOI:** 10.1111/desc.70124

**Published:** 2026-01-05

**Authors:** Kathleen Cracknell, Julia Hauss, Miaofan Chen, Robert Sierp, Lin Bian, Jinjing Wang

**Affiliations:** ^1^ Department of Psychology Rutgers University New Brunswick New Jersey USA; ^2^ Center for Cognitive Science Rutgers University New Brunswick New Jersey USA; ^3^ Department of Human Development and Quantitative Methodology University of Maryland College Park Maryland USA; ^4^ Department of Psychology The University of Chicago Chicago Illinois USA

**Keywords:** Approximate Number System, epistemic trust, estimation, gender bias, gender stereotype, numerical processing

## Abstract

Numerical estimation is one of the key early math skills and predicts children's long‐term math achievement. While children are born with an intuitive “number sense,” they do not form a mapping between nonverbal numerical representations and symbolic numbers until about 5 years of age. This protracted learning process is embedded in children's interactions with other people, although little is known about how children's existing social beliefs may influence how they receive numerical information from other people. Meanwhile, children in the United States develop a stereotype to associate men with being better at math at around 6 years of age. Does this gender stereotype influence how children receive numerical information from other people? Using a numerical estimation task, we showed 5‐ to 7‐year‐old children (*N* = 198; 93 girls; United States) competing answers from a male and a female informant before asking children to provide their own answer. We found that children's answers were biased by the male informant more than the female, even when the female was accurate. This bias was domain‐specific, as there was no difference in children's performance on a memory control task. Furthermore, repeated exposure to inaccurate answers from a male informant (in contrast to accurate answers from a female informant) induced changes in children's estimation even when the informants were no longer present. Therefore, gender stereotypes may have profound impacts on how children acquire and process numerical information.

**Summary:**

- Children form structural mappings between numerical quantities and their mental number line, which can be calibrated by numerical information from other people.
- Here, in two studies, we tested how informant gender influences children's estimation performance.
- Numerical information received from men has stronger calibration effects on children's estimation performance compared to information from women.
- Repeated exposure to overestimation from men has lasting impacts on children's subsequent estimation performance

## 1 Introduction

Accurate acquisition and processing of numerical information is essential for success in the modern world, from understanding financial statements (Park and Cho 2019) to interpreting health statistics (Reyna et al. 2009). While children are born with an intuitive “number sense” to process numerical information (Izard et al. 2009), they have to acquire the ability to map nonverbal numerical representations to symbolic numbers (Le Corre and Carey 2007), a process that is embedded in the social world and largely shaped by surrounding social agents (Silver and Libertus 2022). Thus, sociocultural beliefs, such as cultural stereotypes, may affect children's acquisition and processing of numerical information. In a series of studies, we tested whether informant gender impacts 5‐ to 7‐year‐old children's (*N* = 198) acquisition and processing of numerical information.

### 1.1 Gender Stereotypes Are Early‐Emerging and Could Impact Children's Learning

As early as age six, girls are less likely to attribute high intelligence to their own gender than boys (Bian et al. 2017). At this same age, children are more likely to associate math abilities with boys than with girls (Cvencek et al. 2011; Passolunghi et al. 2014; Steele 2003), even though children typically prefer their own gender in other social contexts (Yang et al. 2022). This type of gender stereotype likely contributes to the later emerging gender gap in students’ participation and achievement in Science, Technology, Engineering, and Mathematics (STEM) fields (Cimpian et al. 2020), as much research has revealed that the gender stereotype about math abilities influences children's self‐concept, motivation, interest, and performance in math (e.g., Ambady et al. 2001; Doyle and Voyer 2016; Cvencek et al. 2015). These gender stereotypes also shape how parents and teachers interact with children (McCoy et al. 2022) and potentially mediate the social transmission of math attitudes within gender groups (Beilock et al. 2010; Gunderson et al. 2012). However, despite the emphasis on mathematics education in our culture, little is known about how gender stereotypes about math may affect young children's acquisition of numerical knowledge.

Children usually prefer to learn from people of the same gender. For example, 4‐ to 6‐year‐old children favor same‐gender informants when learning facts about novel objects (Ma and Woolley 2013) or science (Rackoff et al. 2022), even when the same‐gender informant was pitted against a different‐gender expert (Taylor 2013). Although findings like these typically focus on factual learning, they have been taken to reflect general cognitive mechanisms of children's epistemic trust and selective learning from others (Shafto et al. 2012). However, mathematical learning relies on specific neural circuits (Dehaene et al. 2003) and poses unique challenges for some young learners (Price and Ansari 2013). Moreover, unlike facts, which can be acquired with a single exposure (Markson and Bloom 1997), mathematical knowledge gets built slowly from children's prior knowledge (Wynn 1990). Therefore, it is possible that children's mathematical learning is more susceptible to domain‐specific gender stereotypes. An outstanding question is whether children's gender stereotypes about math can override children's same‐gender preference and result in a stronger bias to acquire numerical information from men versus women.

### 1.2 Numerical Estimation and Calibrating the Mental Number Line

Children's ability to process numerical information relies both on innate cognitive architecture and sociocultural input. Humans and nonhuman species share the ability to approximate quantities, such as distinguishing a flock of 10 birds from 20 birds. This ability is supported by an Approximate Number System (Dehaene 2011), which is available to humans since the first days of life (Izard et al. 2009). However, unlike the ANS, the symbolic number system that allows us to precisely count out 20 birds is unique to humans (Brannon 2005), and it takes children many years to understand the meaning of number words (Carey and Barner 2019).

Number words are ultimately mapped to nonverbal representations, allowing children and adults to estimate quantities by translating between approximate and precise representations (Sieger and Booth 2005). Numerical estimation is one of the key math skills children acquire during early elementary school years (Siegler and Booth 2004) and predicts children's long‐term math outcomes (Geary 2011; Friso‐van den Bos et al. 2015). Children's mastery of these foundational math skills in early childhood is shaped by their social environment, including activities such as labeling quantities with number words (Silver and Libertus 2022). If gender stereotypes can impact the social transmission of numerical knowledge, it may have cascading effects on children's future learning of more advanced math concepts.

Unlike factual knowledge, which is mostly acquired through associative mechanisms, the mapping children construct between the ANS and symbolic numbers is structural in nature (Izard and Dehaene 2008). Children construct a structural isomorphism between their numerical representations (e.g., [dog, dog, dog, dog]) and their mental number line (e.g., 1, 2, 3, 4; Gallistel and Gelman 1992). As a result of this structural mapping, the mental number line can be calibrated easily by numerical input (Izard and Dehaene 2008; Sullivan and Barner 2014). Specifically, when adults and 5‐year‐old children are shown an example set of dots (e.g., 25 dots) and are “calibrated” or told that there are actually 30, both adults and children show biased estimation in the same direction as the calibration. Moreover, this calibration effect does not just influence participants’ estimation of the number they were shown in the example (e.g., saying “thirty” for 25 dots), but also extends to novel quantities across the rest of the mental number line (e.g., saying “sixty” for 50 dots) (Izard and Dehane 2008).

### 1.3 Current Study

The current study is designed based on the calibration effect on children's numerical estimation. But instead of receiving the calibration input from a single female experimenter (Sullivan and Barner 2014), children in the current study received competing input from both a male and a female informant at the same time, following a classic epistemic trust paradigm (Koenig and Harris 2005). Critically, one of the informants responded accurately (e.g., 12), whereas the other informant was obviously overestimating (e.g., 24, which is easily distinguishable from 12 using the ANS; Libertus et al. 2016). Typically, children as young as 3 years endorse information received from accurate over inaccurate informants when learning novel words (Koenig and Harris 2005). However, children's bias to favor men for numerical excellence may override their default preference for accurate informants. If children are biased to place more trust in the male informant's answer, their own estimates should be drawn to the male informant's answer regardless of his accuracy. Alternatively, if children prefer accurate informants or prefer their own gender, they should show no overall bias for the male informant. To test the domain‐specificity of any observed gender bias, children were given a non‐numerical memory task. To test whether the gender effects extend to the rest of children's mental number line, we included a baseline and post‐test block of estimation trials without informant presence.

## 2 Study 1A: Does Informant Gender Bias 5‐ to 6‐Year‐Old Children's Numerical Estimation?

### 2.1 Method

#### 2.1.1 Participants

Sixty‐four children (*M* age = 6;02, 95% CI [5;9, 6;2], range = 5;0–6;11; 32 females) residing in the United States participated in the study. Forty‐one percent of parents identified their child as White; 33% as Asian; 2% as from Hispanic, Latino, or Spanish origins; 2% as Middle Eastern or North African; and the rest identified as multiracial. Ninety‐eight percent of parents reported having a college degree or higher level of education. Reported household income ranged from $10,000 to $200,000+ (*M* = 100,349, SD = $62,110). Children were recruited and tested using the automated algorithm on Children Helping Science (Scott and Schulz 2017). Children were randomly assigned to the two experimental conditions. A power analysis with an assumed medium effect size *f* = 0.25 for the interaction between within‐subject and between‐subject factors (Block × Condition), *alpha* = 0.05, power = 0.95, yielded a total *N* = 54. Sample sizes and analyses were pre‐registered (https://osf.io/wsz9p/registrations).

#### 2.1.2 Materials

Images for the numerical estimation tasks were generated with Matlab Psychtoolbox (Borgo et al. 2012). Images for the informants were selected from the Chicago Face database (Ma et al. 2015) and matched on their overall attractiveness, happiness, trustfulness, and race ratings. All materials are available on OSF (https://osf.io/wsz9p/files/osfstorage).

#### 2.1.3 Procedure

**FIGURE 1:**
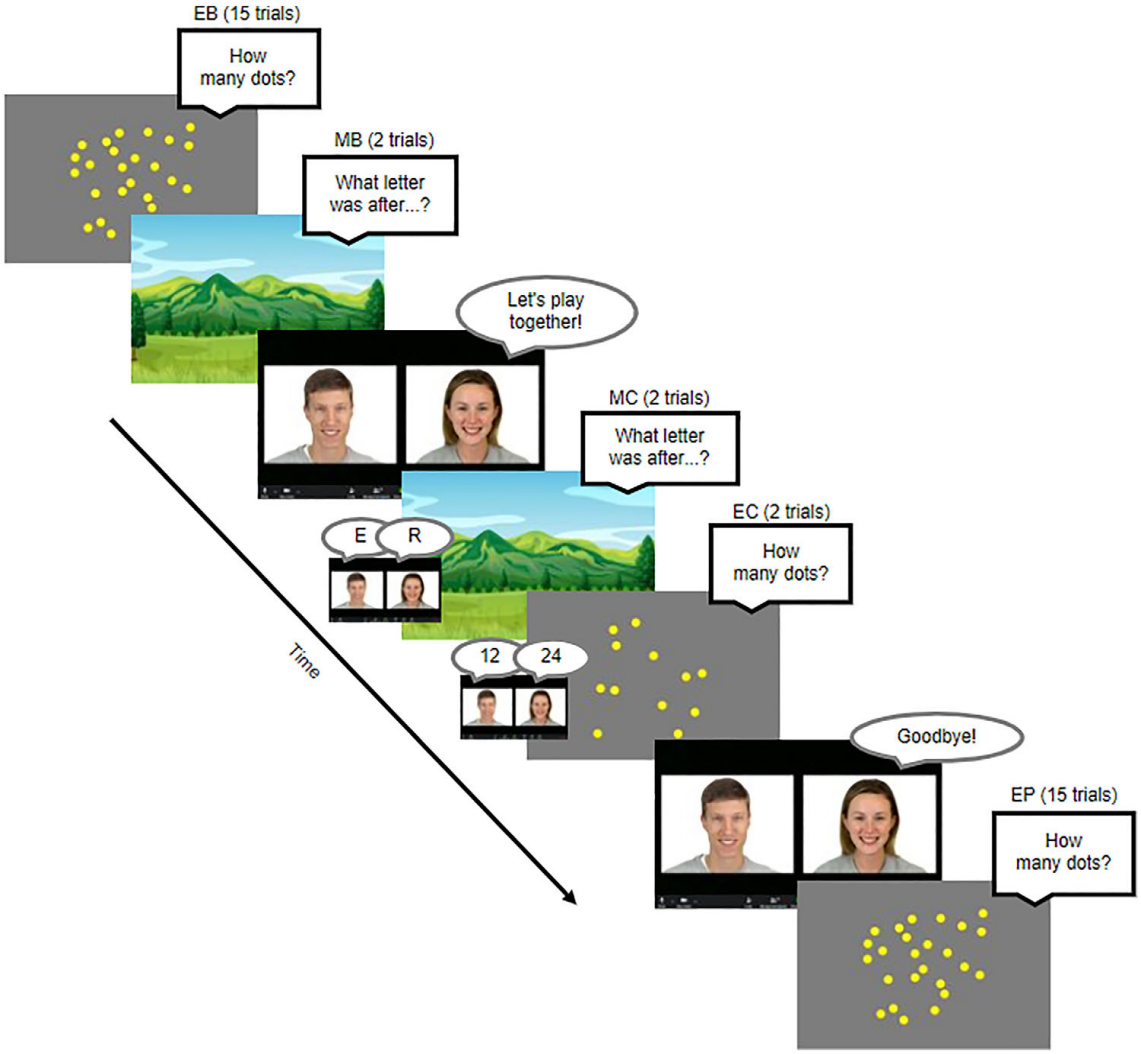
Study 1 succession of tasks (consistent across participants). EB, Estimation Baseline EC, Estimation Calibration; EP, Estimation Post‐test; MB, Memory Baseline; MC, Memory Calibration.

All children completed the Estimation Baseline, Memory Baseline, Memory Calibration, Estimation Calibration, and Estimation Post‐test in the same order (Figure 1). To maintain children's motivation throughout the study, children were told a cover story that they were playing games to help build a zoo. Children earned a new animal for their zoo after every few trials (five for the Estimation tasks and two for the Memory tasks) and heard a fun fact about the animal. Children were asked to say their answer out loud and have their parents help type their responses into the text boxes when prompted. These responses were verified using video recordings. Throughout the study, children were never given feedback on the accuracy of responses provided by themselves or the informants (when present).

**Estimation Baseline**: Children were shown arrays of homogeneously sized dots on the screen and asked to guess aloud how many dots were on the screen without counting. Each numerosity (7, 11,17, 25, and 37 dots) was displayed three times in different configurations following a pseudorandom order. Each dot array was presented for 1500 ms. There were a total of 15 trials. Children were limited to estimations up to 200 for all the Estimation tasks.

**Memory Baseline**: Children were told that they would be playing a memory game and asked to listen closely to some letters. Across two trials consisting of different letter strings, children then heard a list of 10 letters (e.g., N G Z J Q S M L T K) and were prompted to answer about a specific letter (e.g., which letter was after Q?). Letters were recited in 1.5‐s intervals without any visual input. This 10‐item memory task was designed to well‐exceed children's working memory capacity (Gathercole et al. 2004), so that children could not tell what the answer should be.

**Memory Calibration**: The Memory Calibration consisted of two trials that were set up the same way as the Memory Baseline, except that children were told that they were going to play with two other friends (the male and female agents) who would tell children what they thought the answer was first. The order in which the agents provided their responses was counterbalanced. Both agents were always wrong to ensure that there is no information about the reliability of any informant, therefore not inducing any biases in children's epistemic trust prior to the Estimation Calibration.

**Estimation Calibration**: The Estimation Calibration was set up the same way as the Estimation Baseline, and just as in Memory Calibration, children were told that two other friends (the male and female informants) would tell children what they thought the answer was first. Participants were randomly assigned to either the Male‐Overestimate condition or the Female‐Overestimate condition. In the Male‐Overestimate condition, the male informant overestimated (24) while the female informant answered accurately (12); and vice versa for the Female‐Overestimate condition. Participants in both conditions saw the same Calibration trials (two trials of 12 dots in a random configuration). The order in which the agents provided their responses was counterbalanced.

**Estimation Post‐test**: The Estimation Post‐test was identical to the Estimation Baseline with the same set of numerosities in different random configurations. There were 15 trials.

#### 2.1.4 Results

**FIGURE 2:**
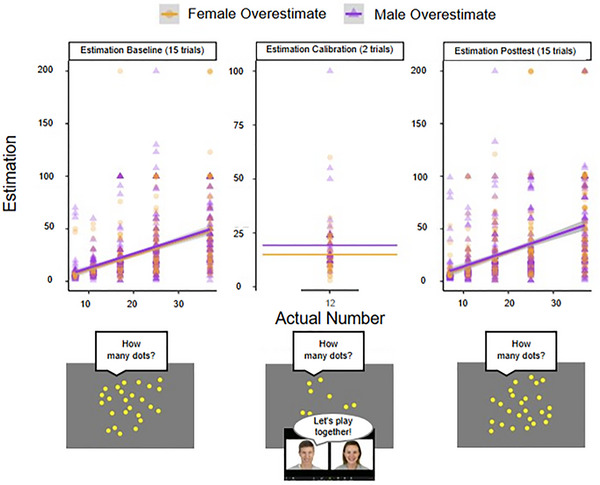
Results of Study 1A showing the relationship between the number of dots viewed and children's corresponding estimations by condition.

We first tested if participants' responses during the Estimation Calibration differed between the Male‐Overestimate and Female‐Overestimate conditions by conducting an independent‐samples *t*‐test. We found a significant difference in children's responses between the Male‐Overestimate (*M* = 19.22, 95% CI [15.72, 22.72]), Female‐Overestimate (*M* = 14.94, 95% CI [12.69, 17.19]) conditions, *t*(52.90) = −2.09, *p* = 0.040, *d* = −0.39 (Figure 2).

To test the domain‐specificity of the observed gender bias, we asked if participants' responses differed in the Memory Calibration task between the two conditions. Responses were coded into the following three categories: same as female, same as male, or different from either informant. There were no significant differences in the Memory Calibration task responses between the two conditions, *X* ^2^ (2, *N* = 64) = 1.59, *p* = 0.449, OR = 2.50.

We then asked if the Estimation Calibration had any lasting effects on children's numerical estimation by conducting a linear mixed‐effects model on participants' responses in the Estimation Baseline and Post‐test, predicting Estimates for each trial with Target Number, Block (Baseline or Post‐test), Condition (Male Overestimate and Female Overestimate), and Age as fixed effects, and Participant as random intercept. The mixed‐effects models were fitted using the lme4 package (Bates et al. 2015) in R (version 4.1.1; R Core Team 2020). Models were fit using restricted maximum likelihood estimation (REML). We obtained *p* values using likelihood‐ratio tests comparing the full model with the effect in question and the model without the effect in question. Consistent with previous research (Sullivan and Barner 2014), children's estimation increased significantly as Target Number increased, *X* ^2^(1) = 33.82, *p* < 0.001, OR = 3.50. We found no significant main effects of Block, Condition, Age, or any interaction terms, *X* ^2^s < 0.42, *ps* > 0.51.

We also used maximum likelihood estimates (MLEs) to estimate each participant's coefficient of variance (CV) and slope in the Estimation Baseline and Estimation Post‐test. This model assumes that each participant's Responses (R) in each Block is a power function of the Target Number (*n*) (Izard and Dehaene 2008; Sullivan and Barner 2014). The log‐transformed responses, therefore, are drawn from a normal distribution log(*R*)∼(log(*α*) + *β*log(*n*), γ), where *β* is the slope and *γ* is CV. The MLE models were fitted using the bbmle package (Bolker and Bolker 2017). We then fitted a separate repeated‐measures ANOVA to test if the Estimation Calibration caused any changes in children's CV and slope, with Block (Baseline or Post‐test) as a within‐subject factor and Condition (Male Overestimate or Female Overestimate) as a between‐subject factor. We found no significant effects or interactions, *F*s < 3.36, *p*s > 0.72.

#### 2.1.5 Discussion

Consistent with our hypothesis, Study 1A found that there is an effect of informant gender on children's immediate numerical estimation. Specifically, when children were presented with competing input from male and female informants, their immediate numerical estimations were biased toward the male informant's response. The bias was likely specific to the numerical domain, as it was not observed during the memory task. However, we observed no lasting effects on children's performance on the Estimation post‐test. One possibility is that Study 1A did not have a sufficient sample size to capture the hypothesized lasting gender effects on children's numerical estimation. Relatedly, factors such as participants’ own gender and baseline numerical estimation skills may moderate how they respond to the estimation calibration trials (e.g., children with better numerical estimation skills may find the calibration of 12 dots as “24” more unacceptable). Along with the pre‐registered sample of *N* = 64 5‐ to 6‐year‐old children reported here, we accidentally collected data from a slightly wider age range and larger sample size due to an experimenter oversight. This larger accidental sample granted us an opportunity to explore the possibility that there may be stronger effects of informant gender on children's numerical epistemic trust, or that these effects may be moderated by participants’ own gender and numerical skills. Below in Study 1B, full sample results and these additional analyses are reported, which should be interpreted as exploratory.

## 3 Study 1B: Does Informant Gender Bias 5‐ to 7‐Year‐Old Children's Numerical Estimation?

### 3.1 Method

#### 3.1.1 Participants

Ninety‐six children (*M* age = 6;4, 95% CI [6;2, 6;5], range = 5;1–7;11; 50 females) residing in the United States participated in the study (64 of these participants were included in the analysis in Study 1A). Forty‐seven percent of parents identified their child as White; 22% as Asian; 5% as from Hispanic, Latino, or Spanish origins; 1% as Middle Eastern or North African; 2% as other; and the rest identified as multiracial. All parents reported having a college degree or higher level of education. Reported household income ranged from $10,000 to $200,000+ (*M* = 103,803, SD = $60,193). Children were recruited and tested using the automated algorithm on Children Helping Science (Scott and Schulz 2017). Children were randomly assigned to the two experimental conditions.

#### 3.1.2 Materials and Procedure

The materials and procedure are the same as those used in Study 1A.

#### 3.1.3 Results

**FIGURE 3:**
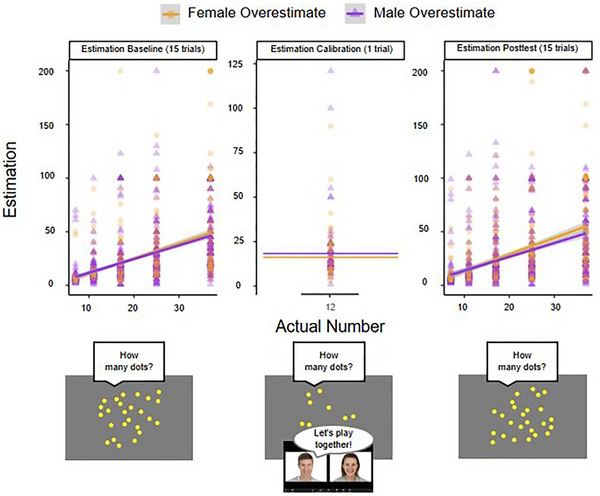
Results of Study 1B showing the relationship between the number of dots viewed and children's corresponding estimations by condition.

We first tested if participants' responses during the Estimation Calibration differed between the Male‐Overestimate and Female‐Overestimate conditions by conducting an independent‐samples *t*‐test. Contrary to the significant effect observed from the smaller sample from Study 1A, we found no significant difference in children's responses between the Male‐Overestimate (*M* = 18.31 95% CI [15.30, 21.32]), Female‐Overestimate (*M* = 16.14, 95% CI [13.70, 18.57]) conditions, *t*(186.91) = 1.11, *p* = 0.267, *d* = 0.16 (Figure 3).

To explore why the effect disappeared with the larger sample, we conducted an unregistered ANOVA with children's responses during the Estimation Calibration as dependent variable, Condition (Male‐Overestimate vs. Female‐Overestimate) and participant Gender (Male vs. Female) as between‐subject factors, and participants’ Baseline Estimates as covariate. We found a significant effect of Baseline Estimates, *F*(1, 92) = 13.99, *p* = 0.004, *η_p_^2^*  = 0.996, and no other effects, *p*s > 0.096.

We asked if participants' responses differed in the Memory Calibration task between the two conditions. Responses were coded into three categories: same as female, same as male, or different from either informant. There were no significant differences in the Memory Calibration task responses between the two conditions, *X* ^2^ (2, *N* = 96) = 0.50, *p* = 0.780, OR = 0.76.

We then asked if the Estimation Calibration had any lasting effects on children's numerical estimation by conducting the same linear mixed‐effects model as in Study 1A on participants' responses in the Estimation Baseline and Post‐test, predicting Estimates for each trial with Target Number, Block (Baseline or Post‐test), Condition (Male Overestimate and Female Overestimate), and Age as fixed effects, and Participant as random intercept. As in Study 1A, children's estimation increased significantly as Target Number increased, *X* ^2^(1) = 136.96, *p* < 0.001, OR = 5.21. There were no other significant effects, *X* ^2^s < 4.45, *ps* > 0.071.

**FIGURE 4:**
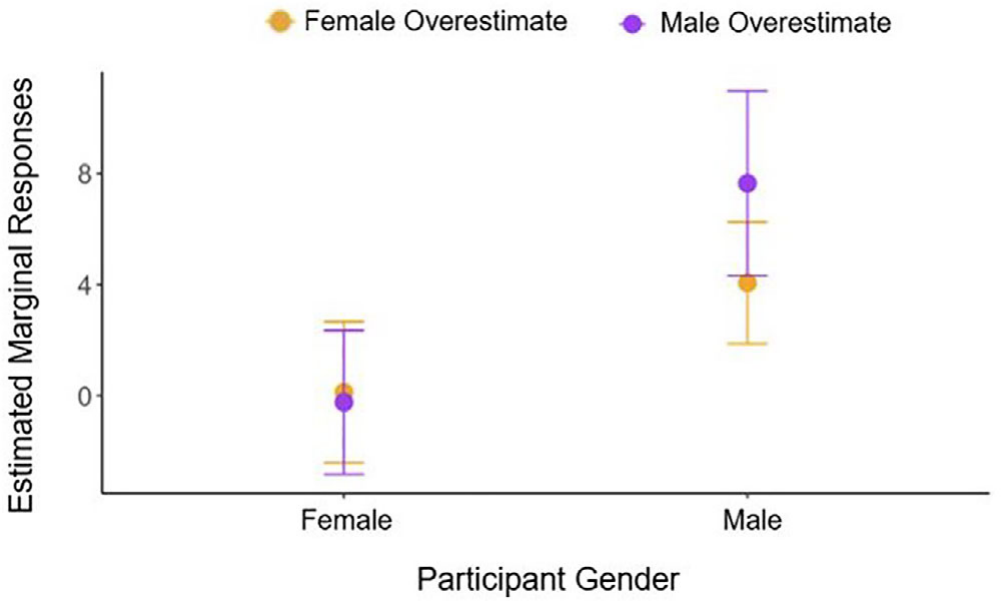
Exploratory results of Study 1B showing the effect of a change in children's own gender on the predicted estimation responses as a function of Block and Condition.

Since previous research suggested that children may prefer informants of their own gender, we also explored children's own Gender as an additional fixed effect in this model. This analysis revealed a significant Condition × Block × Gender interaction, *X* ^2^(2) = 14.80, *p* < 0.001, OR Baseline = 1.01, OR Post‐test = 1.40. Using the marginal effects package in R (Arel‐Bundock et al. 2024), we computed pairwise post hoc contrasts to examine the estimated effects of changing each of these variables (e.g., Gender) in relation to the remaining two variables (e.g., Block and Condition), and found that the only significant contrast was the Condition‐Gender contrast, *p* = 0.002, as visualized in Figure 4. This pattern suggests that girls may be less susceptible to false information provided by unknown informants, given the current paradigm.

As in Study 1A, we also used MLEs to estimate each participant's CV and slope in the Estimation Baseline and Estimation Post‐test. We found no significant effects or interactions, *F*s < 1.54, *p*s > 0.218. Including participant gender in these models revealed no significant effects, *p*s > 0.227.

#### 3.1.4 Discussion

In Study 1B, we expanded the age range to 5‐ to 7‐year‐olds and increased our sample size by 50% to test whether the lack of lasting effects from Study 1A was due to our small age range and sample size. Contrary to our predictions, in this larger sample, children on average did not show any bias in their numerical estimation when the gender of the informant differed. Moreover, exploratory analyses revealed that children's own gender and their baseline estimation accuracy may have moderated these effects. However, our study was not specifically designed to test the roles of children's own gender or their baseline estimation accuracy in how they respond to different gendered informants, limiting the robustness of these results. Future research should specifically test the roles of children's own gender and cognitive skills in moderating their epistemic trust in numerical information from different informants.

In both Studies 1A and Study 1B, we found no significant effect of the Calibration Condition on children's performance on the Estimation Post‐test, in which the informants were no longer present. It is possible that informant gender only influences children's immediate estimation response and cannot have any lasting impacts on their numerical processing. Alternatively, children may need more than two exposures from the informants’ answers to experience lasting changes in their estimation. Relatedly, the gap between the wrong answer (24) and the correct answer (12) may be too large for children (especially those with accurate baseline numerical estimation themselves) to believe in the informants.

Study 2A tests this possibility by providing children with 10 Estimation Calibration trials with a range of different target numbers and responses that vary in their distance on the mental number line. If informant gender can only bias children's immediate numerical estimation, we should continue to see no changes in children's estimation performance. If repeated calibration from male informants causes more lasting impacts on children's estimation, we may see significant changes in children's estimation performance. In addition, Study 2A asks whether these group‐level differences are linked to differences in children's gender belief biases, measured by children's beliefs about whether one gender is more nice or numerically competent than the other.

## 4 Study 2A: Does Informant Gender Bias Have Lasting Impacts on 5‐ to 6‐Year‐Old Children's Estimation Performance?

### 4.1 Method

#### 4.1.1 Participants

Sixty‐four children (*M* = 6;1, 95% CI [5;9, 6;2] years, range = 5;0–6;11 years; 32 females) residing in the United States participated in the study. None of the participants in Study 2A participated in Study 1A or 1B. Sixty‐five percent of parents identified their child as White, 22% as Asian, and the rest identified as multiracial. All but 1.6% of all parents reported having a college degree or higher level of education. Reported household income ranged from $10,000 to $200,000+ (*M* = 116,792, SD = $54,377). Children were recruited and tested using the automated algorithm on Lookit (Scott and Schulz 2017). Children were randomly assigned to the two experimental conditions. Sample sizes and analyses were preregistered (https://osf.io/wsz9p/registrations).

#### 4.1.2 Materials and Procedure

The materials and procedure were the same as in Study 1A except for the following:

For the Estimation Calibration trials, the smiling agents from Study 1A were replaced with similar agents with a neutral expression. Due to an experimenter oversight, Study 1A selected smiling instead of neutral faces, which may not have perfectly matched their average attractiveness, happiness, and trustworthiness ratings (Ma et al. 2015). Therefore, we selected a set of neutral faces for Study 2A to more closely match the stimuli along these dimensions. This change helps to test the robustness of our results while minimizing the influence of emotion on children's responses.

To potentially increase the impact of the informants on children's estimation performance, we increased the number of Estimation Calibration trials from two in Studies 1A and 1B to 10 in Study 2A. Array sizes of 12, 24, 36, 48, and 60 were presented in a pseudo‐random order for all participants. One of the agents always provided accurate estimates for all the trials, whereas the other agent (the male in the Male‐Overestimate condition and the female in the Female‐Overestimate condition) always provided an answer that was 12 above the actual quantity (e.g., saying 24 for 12 dots or saying 72 for 60 dots). Although the absolute distance between the overestimation and the actual numerosity was always 12, the ratio between the two decreased as the array size increased, making them less distinguishable.

To explore potential mechanisms of the gender effects on children's estimation performance, we also measured children's gender beliefs (Shu et al. 2022). For each item, children were shown a novel pair of male and female agents and were asked to decide which of the two agents was “really, really nice” (two trials), “really, really good at math” (two trials), or “really, really good at the dots guessing game” they had just played (two trials).

**FIGURE 5:**
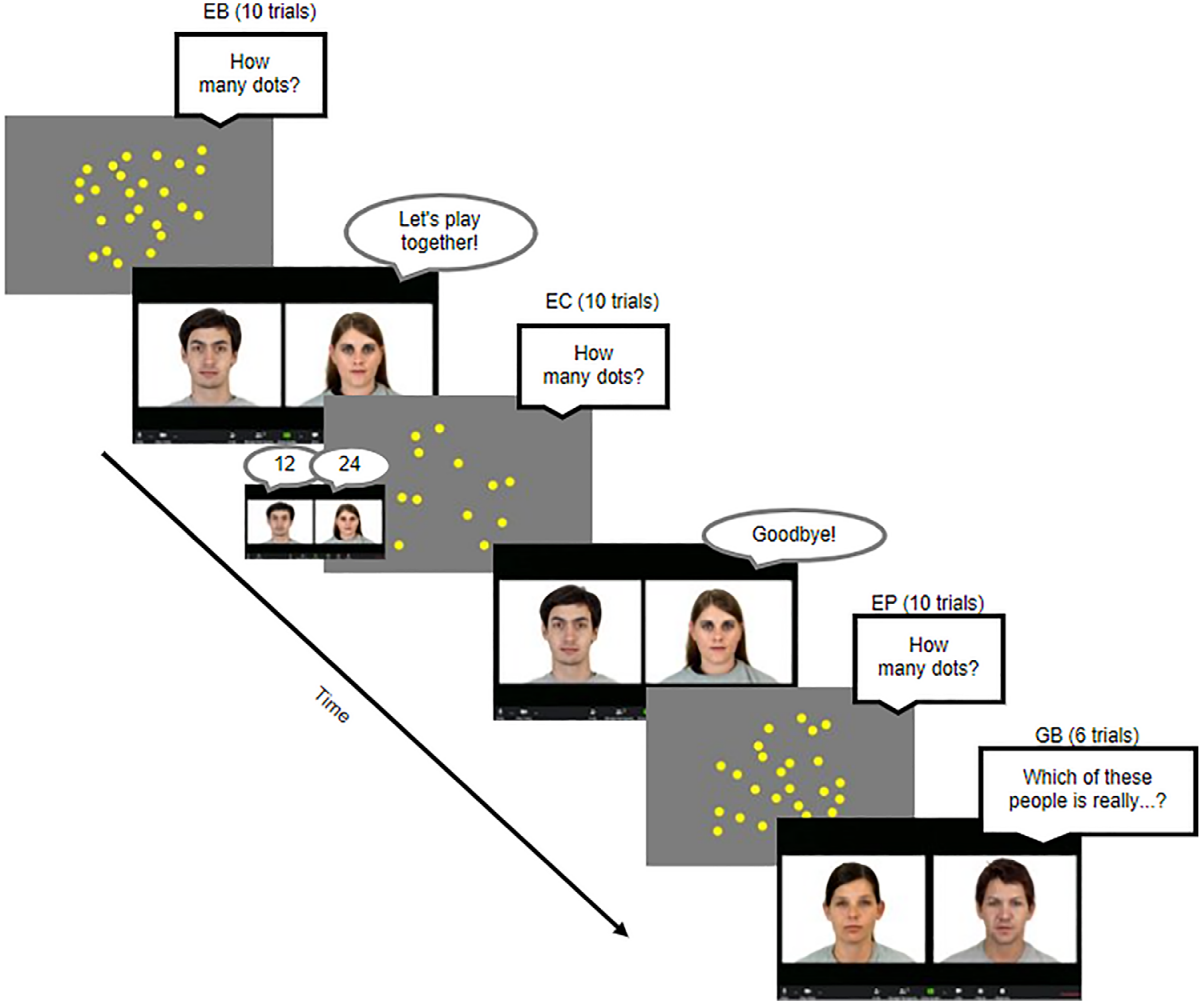
Study 2A succession of tasks (consistent across participants). EB, Estimation Baseline; EC, Estimation Calibration; EP, Estimation Post‐test; GB, gender beliefs assessment.

To keep the study within a reasonable length, we reduced the Estimation Baseline and Estimation Post‐test to 10 trials each (each numerosity displayed twice instead of three times) and removed the Memory Baseline and Memory Calibration tasks (Figure 5).

#### 4.1.3 Results

**FIGURE 6:**
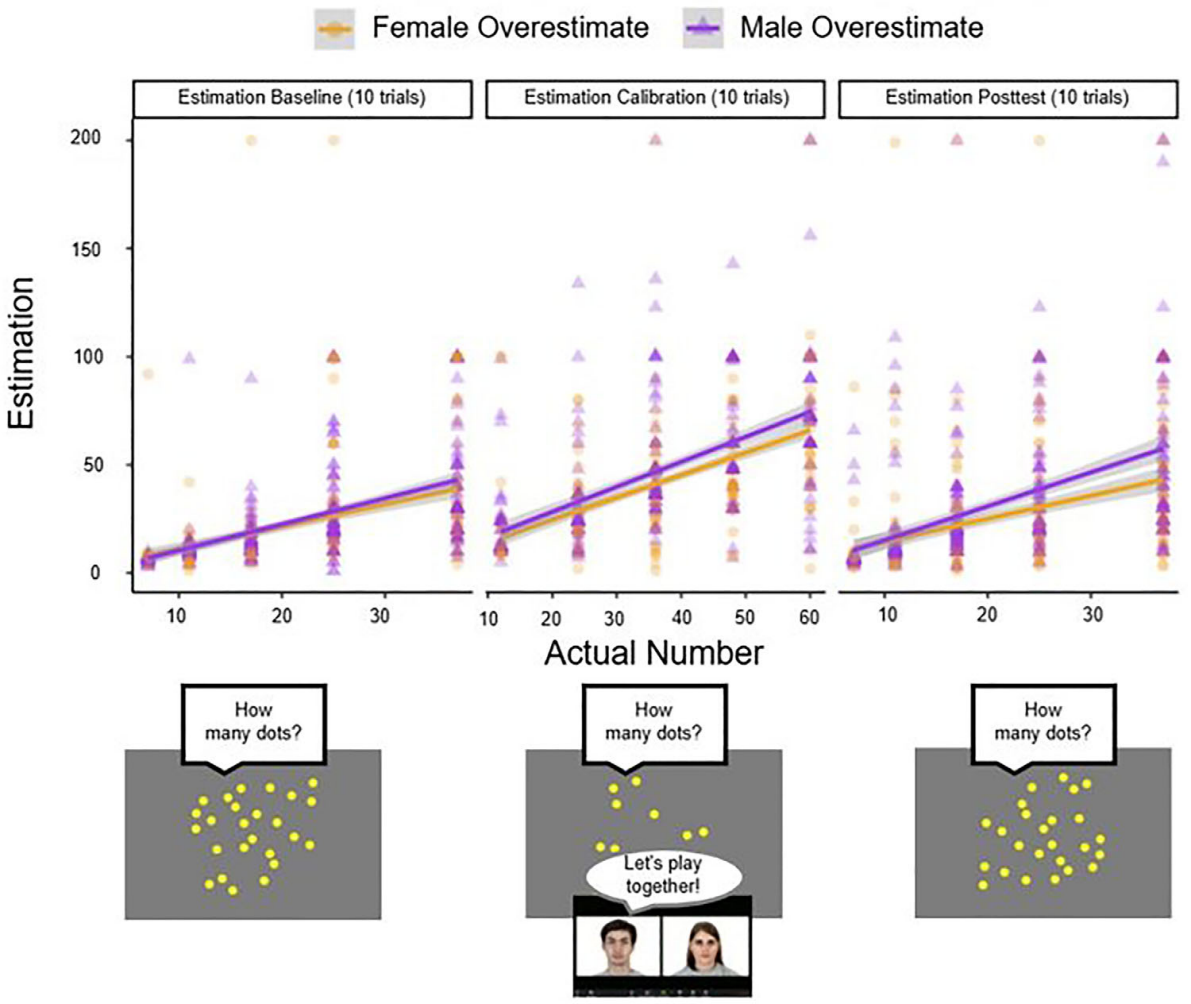
Results of Study 2A showing the relationship between the number of dots viewed and children's corresponding estimations by condition.

As in Study 1A, we first conducted an independent‐samples *t*‐test comparing children's responses in the Estimation Calibration trials between the Male‐Overestimate and Female‐Overestimate conditions. Similar to Study 1A, we found a significant difference in children's responses between the Male‐Overestimate (*M* = 46.72, 95% CI [43.14, 50.30]), Female‐Overestimate (*M* = 41.16, 95% CI [38.14, 44.17]) conditions, *t*(618.68) = 2.34, *p* = 0.019, *d* = 0.18 (Figure 6).

We then asked if Estimation Calibration had any lasting effects on children's numerical estimation by conducting a linear mixed‐effects model on participants' responses in the Estimation Baseline and Post‐test, predicting Estimates for each trial with Target Number, Block (Baseline or Post‐test), Condition (Male Overestimate or Female Overestimate) and Age as fixed effects, and Participant as random intercept. We found a significant main effect of Target Number, *X* ^2^(1) = 105.85, *p* < 0.001, OR = 2.95 and Target Number × Block × Condition interactions, *X* ^2^(3) = 16.47, *p* < 0.001, OR = 1.60.

As in Study 1A, we used MLEs to model each participant's CV and slope in the Estimation Baseline and Estimation Post‐test. We then fitted a separate repeated‐measures ANOVA for CV and slope, with Block (Baseline or Post‐test) as a within‐subject factor and Condition (Male Overestimate or Female Overestimate) as a between‐subject factor. We found a significant effect of Block on CV, *F*(1, 62) = 4.98, *p* = 0.029, *η_p_* ^2^  = 0.074, and no other significant effects.

We asked if participants' responses differed in the follow‐up task between the two conditions (Male‐Overestimate vs. Female‐Overestimate), and whether participants in either condition showed bias towards thinking that males are better at estimating and math, relative to being nice. The response distributions were compared between conditions using chi‐square and compared to chance using exact tests. There were no significant differences between Conditions and thinking that males are better at estimating and math in relation to being nice, χ^2^(4, *N* = 64) = 2.13, *p* = 0.711, Cramér's *V* = 0.18, We followed up with a Fisher's exact tests indicating that the response distribution is not above chance, *p*s > 0.721.

#### 4.1.4 Discussion

Similar to Study 1A, Study 2A found a math‐gender bias such that children's immediate numerical estimation was biased by the gender of the informant. Additionally, there was a lasting effect on children's later numerical estimation, supporting our hypothesis that repeated and potentially more believable numerical information from male informants could cause lasting impacts on numerical estimation. However, these effects were not reflected clearly in children's slope or CV estimates. One possibility is that our study was underpowered to detect mechanistic changes in different aspects of children's numerical representations (i.e., how their mental representations are aligned, as measured by slope, vs. how much internal noise they have, as measured by CV). It is also possible that there is a lot of individual variability in how children's numerical representations are influenced by the estimation calibration, which may result in a lack of group‐level effect. Study 2B will use an accidental larger sample to test the first possibility.

One likely candidate for explaining individual differences in children's responses to the estimation calibration is their gender beliefs. To ask whether children's gender beliefs may be linked to the gender bias in children's response to numerical information from different informants, we also measured children's beliefs about how nice or good at numerical tasks each gender should be, and found that children's gender beliefs did not differ across the Estimation Calibration conditions, suggesting that gender belief differences could not explain the group level effects of informant conditions. Although Study 2A found no group‐level differences when analyzing the gender beliefs separately for being nicer or for being better at numerical tasks, it remains possible that there are individual differences in the relative strength of children's beliefs between thinking which gender is nicer versus which gender is better at numerical tasks. These relative differences may be linked to children's estimation performance in ways that are more subtle than a simple main effect of condition. To explore these possibilities, Study 2B also computed a Numerical‐Gender Bias Score (controlled for Nice‐Gender Bias) for each participant that captures the relative strength of their gender belief for males being better at numerical tasks relative to males being nicer. We characterized how these domain‐specific gender bias scores varied between the two Estimation Calibration conditions, as well as exploring how they relate to how much children were biased in their numerical estimation across conditions. In addition to the pre‐registered sample of *N* = 64 5‐ to 6‐year‐old children reported here, we accidentally collected data from a slightly wider age range and larger sample size due to an experimenter oversight. This larger accidental sample granted us an opportunity to explore the possibility that there may be more subtle links between children's gender beliefs and their numerical epistemic trust. Below in Study 2B, full sample results and these additional analyses are reported, which should be interpreted as exploratory.

## 5 Study 2B: Does Informant Gender Bias Have Lasting Impacts on 5‐ to 7‐Year‐Old Children's Estimation Performance?

### 5.1 Method

#### 5.1.1 Participants

One hundred and two children (*M* = 6;37, 95% CI [6;2, 6;5] years, range = 5;0–7;8 years; 43 females) residing in the United States participated in the study (64 of these participants were included in the analysis in Study 2A). Sixty‐three percent of parents identified their child as White, 2% as Black, 17% as Asian, 1% as from Hispanic, Latino, or Spanish origins, 1% as Middle Eastern or North African, and the rest identified as multiracial. All but 1% of all parents reported having a college degree or higher level of education. Reported household income ranged from $10,000 to $200,000+ (*M* = 119,824, SD = $54,894). Children were recruited and tested using the automated algorithm on Children Helping Science (Scott and Schulz 2017). Children were randomly assigned to the two experimental conditions.

#### 5.1.2 Materials and Procedure

The materials and procedure were the same as in Study 2A. In addition to analyzing the gender belief responses as raw scores, we also tried to quantify potential links between children's numerically specific gender beliefs and their gender biases in numerical epistemic trust. To have a single measure that reflects children's domain‐specific gender bias in the context of the numerical estimation task, we computed a Numerical‐Gender Bias Score (controlled for Nice‐Gender Bias) based on the gender belief measures: We first computed the average of the “math” and “dots” questions. We then subtracted “nice” ratings from the “numerical” ratings to account for domain‐general gender preferences. To put everything on an interpretable −1∼1 scale, we divided the difference score by their sum, following common practice in infant research to normalize responses to be suitable for parametric analyses (e.g., Csibra et al. 2016).

#### 5.1.3 Results

**FIGURE 7:**
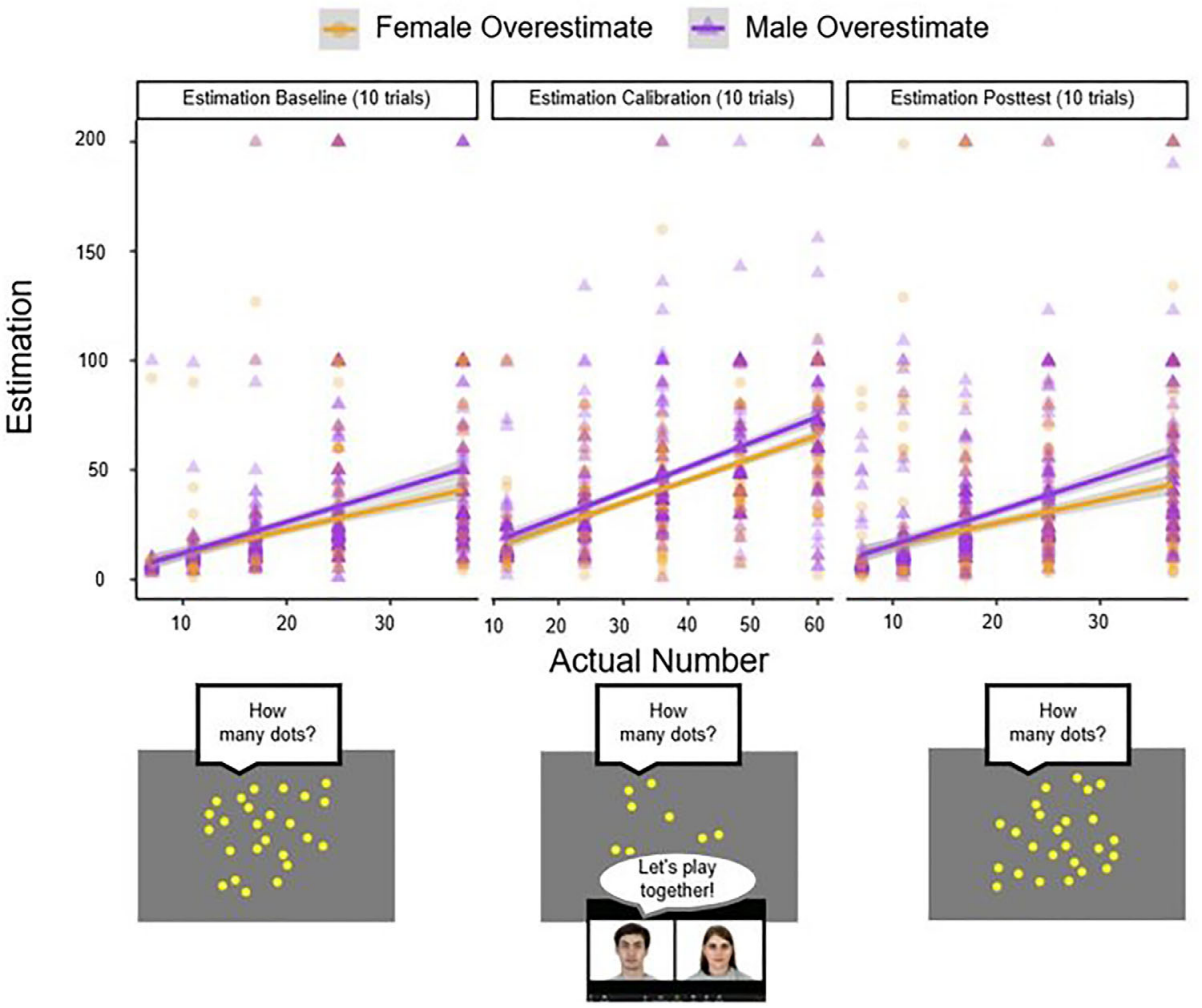
Results of Study 2B showing the relationship between the number of dots viewed and children's corresponding estimations by condition.

As in the previous Experiments, we first conducted an independent‐samples *t*‐test comparing children's responses in the Estimation Calibration trials between the Male‐Overestimate and Female‐Overestimate conditions. Similar to Studies 1A and 2A, we found a significant difference in children's responses between the Male‐Overestimate (*M* = 46.74, 95% CI [44.04, 49.43]), Female‐Overestimate (*M* = 41.15, 95% CI [38.80, 43.49]) conditions, *t*(1010.3) = 3.07, *p* = 0.002, *d* = 0.19 (Figure 7).

These effects were not moderated by participants’ own gender, as there was no Gender × Condition interaction when entering both Gender and Condition as between‐subject factors in an ANOVA, *F*(1, 98) = 0.13, *p* = 0.714, *η_p_* ^2^ = 0.001.

All findings remain when excluding children's responses that fell out of three standard deviations from the group average in each block. With outliers removed, there remains a significant difference in children's responses between the Male‐Overestimate (*M* = 44.39, 95% CI [42.10, 46.68]), Female‐Overestimate (*M* = 40.23, 95% CI [38.12, 42.34]) conditions, *t*(1003.1) = 2.62, *p* = 0.009, *d* = 0.16. There remained no moderation of participants’ own gender as above using the same model when outliers were removed, *F*(1, 96) = 0.13, *p* = 0.714, *η_p_* ^2^ = 0.002.

We then asked if Estimation Calibration had any lasting effects on children's numerical estimation by conducting a linear mixed‐effects model on participants' responses in the Estimation Baseline and Post‐test, predicting Estimates for each trial with Target Number, Block (Baseline or Post‐test), Condition (Male Overestimate or Female Overestimate), and Age as fixed effects, and Participant as a random intercept. We found a significant main effect of Target Number, *X* ^2^(1) = 122.36, *p* < 0.001, OR = 2.86, and Target Number × Block × Condition interactions, *X* ^2^(3) = 17.56, *p* = < 0.001, OR = 1.60.

Exploratory analysis, including children's own Gender as an additional fixed effect in this model, revealed the same pattern of results, with a significant Condition × Target Number × Block interaction, *X* ^2^(5) = 27.29, *p* < 0.001, but no significant interaction with Gender, *X* ^2^(6) = 3.06, *p* = 0.805.

As in Study 2A, we used MLEs to model each participant's CV and slope in the Estimation Baseline and Estimation Post‐test. We then fitted a separate repeated‐measures ANOVA for CV and slope, with Block (Baseline or Post‐test) as within‐subject factor and Condition (Male Overestimate or Female Overestimate) as between‐subject factor. Unlike previous experiments, we found a significant main effect of Condition for CV, *F*(1, 100) = 5.01, *p* = 0.027, *η_p_* ^2^  = 0.048, and no other significant effects. However, since there was no significant Block × Condition interaction, these results may be partially due to pre‐existing differences in children's CV in the sample.

We then asked if participants' responses differed in the follow‐up gender belief task between the two conditions (Male‐Overestimate vs. Female‐Overestimate), and whether participants in either condition showed bias towards thinking that males are better at estimating and math, relative to being nice. The response distributions were compared between conditions using chi‐square and compared to chance using exact tests. Similar to Study 2A, responses did not differ between the Conditions in asking if children showed a bias in thinking that males are better at estimating and math relative to being nice, χ^2^(4, *N* = 102) = 0.03, *p* = 0.999, Cramér's *V* = 0.13, We followed up with a Fisher's exact tests indicating that the response distributions are not above chance, *p*s > 0.721.

We then conducted a series of exploratory analyses to understand the computed Numerical‐Gender Bias Scores (controlled for Nice‐Gender Bias). Consistent with the above nonparametric analyses, there was no difference in Numerical‐Gender Bias Scores (controlled for Nice‐Gender Bias) in the Male‐Overestimate (*M* = 0.15, 95% CI [−0.02, 0.32]) and Female‐Overestimate (*M* = 0.31, 95% CI [0.13, 0.48]) conditions, *t*(99.56) = −1.30, *p* = 0.195, *d* = −0.13. However, children in the Female‐Overestimate (*t*(47) = 3.55, *p* < 0.001, *d* = 0.51), but not the Male‐Overestimate condition (*t*(53) = 1.72, *p* = 0.091, *d* = 0.23), were significantly biased to think that males were better at math.

**FIGURE 8:**
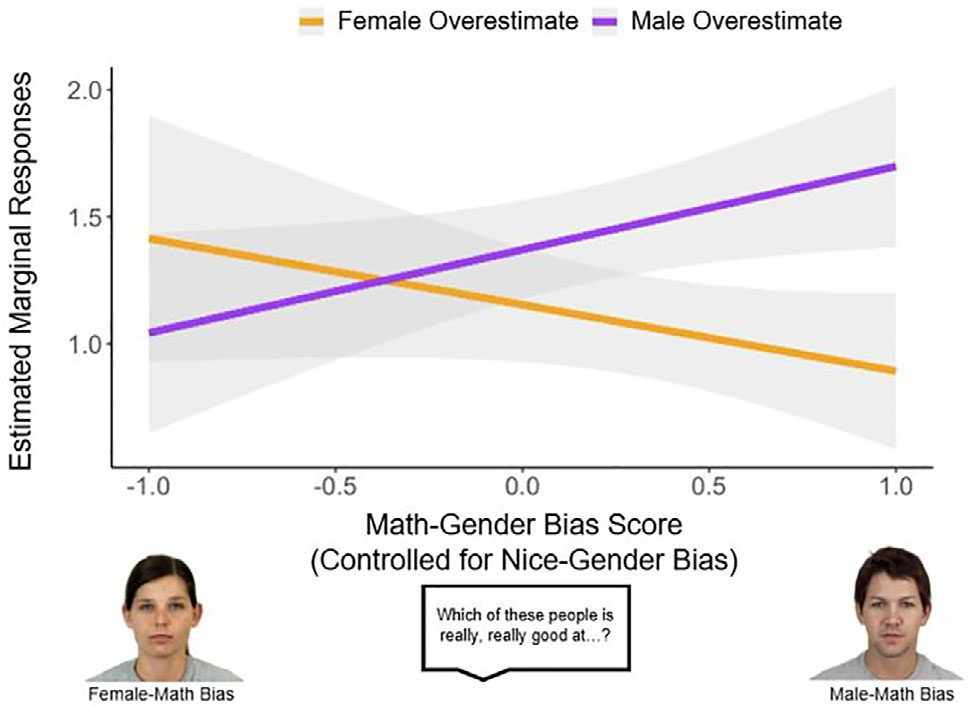
Exploratory results of Study 2B showing the effect of a change in children's Numerical Gender Bias Score (controlled for Nice‐Gender Bias) on the predicted estimation responses as a function of Target Number and Condition.

To explore how these differences in children's numerical gender biases relate to children's numerical epistemic trust, we conducted a mixed‐effects model on participants' responses in the Estimation tasks, predicting Estimates for each trial with Condition (Male Overestimate or Female Overestimate), Block (Pretest or Post‐test), Target Number, and Numerical Gender Bias Score as fixed effects, and Participant as random intercept. We found a significant Target Number × Condition × Numerical Gender Bias Score interaction, *X* ^2^(1) = 6.48, *p* = 0.010, OR = 0.55. Using the marginal effects package in R (Arel‐Bundock et al. 2024), we computed pairwise post hoc contrasts to examine the estimated effects of changing each of these variables (e.g., Numerical Gender Bias Score) in relation to the remaining two variables (e.g., Target Number and Condition), and found that the only significant contrast was the Estimate‐Numerical Gender Bias Score contrast, *p* = 0.013, as visualized in Figure 8. Children in the Male‐Overestimate condition showed an increasing change in their estimation responses as their Numerical Gender Bias and Target Number increased, whereas children in the Female‐Overestimate condition showed the reversed pattern. Since the male informant in these two conditions provided responses in the opposite directions (i.e., providing larger numbers in Male Overestimate and providing smaller numbers in Female Overestimate), this pattern suggests that children with stronger numerical gender bias were more likely to be influenced by the male informant, especially when the target number became larger.

## 6 General Discussion

Research on children's and adults’ gender stereotypes has provided important insights into the origins of gender gaps in STEM participation and achievement (Bian 2022; Cvencek et al. 2011; Sadler et al. 2012). However, to date, there has been little experimental evidence on how gender stereotypes contribute to children's acquisition of mathematical knowledge from adults. Inspired by findings from both social cognition and numerical cognition, the current work tested how children's numerical estimation responds to competing numerical information received from men and women. Across two sets of studies, we found that 5‐ to 7‐year‐old children's estimation responses were biased more by the male informant's answer over the female informant's answer, even when the male was inaccurate, overriding children's default preference to place more epistemic trust in reliable and same‐gender informants (Rackoff et al. 2022). These effects were specific to the numerical domain, as there was no difference in children's memory task performance following the informants’ answers.

Furthermore, Studies 2A and 2B showed that repeated exposure to numerical information from a male informant, in contrast to a female informant, influences children's performance in a subsequent estimation task even though the informants were no longer present. This effect suggests that the male bias has a lasting influence on children's acquisition of numerical knowledge. Past research found that children consider informants’ knowledgeability and reliability when deciding who to trust and learn from (Pesch and Koenig 2018; Landrum et al. 2016). However, in the numerical domain, children appear to override their own numerical knowledge according to information received from a male informant when it diverged from a female informant's accurate estimates.

We also found that children's gender stereotype beliefs moderated these effects, with children in the Male‐Overestimate condition who endorse stronger male‐numerical links (relative to male‐nice links) showing stronger effects of the estimation condition on their estimation responses, especially when the target number became larger. These results suggest that gender beliefs may play a role in shaping children's numerical processing. However, these results are correlational in nature, leaving the causal direction unclear. In particular, the current study only measured children's gender‐math beliefs after the estimation tasks. Therefore, it remains possible that children's experience with the informants shaped their gender beliefs rather than vice versa. Specifically, children in the Female‐Overestimate condition, but not the Male‐Overestimate condition, showed a significant bias to think that males are better at numerical tasks relative to being nice, suggesting that children may be more sensitive to misinformation provided by females when updating their gender‐specific beliefs. Future investigations of the causal links between gender‐math beliefs and children's responses to different gendered informants will provide more insights into the developmental and cognitive mechanisms underlying gender gaps in mathematical fields.

Taken together, results from the current study suggest that informant gender influences how children receive numerical information from adults. Specifically, numerical information provided by a male informant has a stronger impact on children's estimation performance over competing information provided by a female informant, even when the male informant is wrong. These findings have cross‐disciplinary implications for understanding how children learn numerical information from other people in the real world. Given how numerical estimation is essential for correctly interpreting economical and medical information (Reyna et al. 2009; Park and Cho 2019), people's stereotypes about who is good at math may not only influence their own STEM engagement, but also their reception of important real‐world information that could directly influence their own well‐being. Our study provides the theoretical and methodological foundation for future investigations of these important issues.

## Funding

The authors have nothing to report.

## Conflicts of Interest

The authors declare no conflicts of interest.

## Data Availability

All data are publicly available (https://osf.io/wsz9p/files/osfstorage).
